# A protocol for measuring the impact of a smoke-free housing policy on indoor tobacco smoke exposure

**DOI:** 10.1186/s12889-019-7043-3

**Published:** 2019-05-30

**Authors:** Rodrigo Arce Cardozo, Alexis Feinberg, Albert Tovar, M. J. Ruzmyn Vilcassim, Donna Shelley, Brian Elbel, Sue Kaplan, Katarzyna Wyka, Ana M. Rule, Terry Gordon, Lorna E. Thorpe

**Affiliations:** 1 Department of Population Health, New York University School of Medicine, 180 Madison Avenue, New York, NY 10016 USA; 2 Graduate School of Public Health and Health Policy, City University of New York, New York, NY 10027 USA; 3 Department of Environmental Health and Engineering, Johns Hopkins Bloomberg School of Public Health, 615N Wolfe Street, Baltimore, MD 21205 USA; 4 Department of Environmental Medicine, New York University School of Medicine, 341 East 25th Street, New York, NY 10010 USA

**Keywords:** Public housing, Tobacco smoke pollution, Smoke free housing, Multi-unit housing, Air monitoring, protocol

## Abstract

**Background:**

Tobacco remains a leading cause of preventable death in the U.S., responsible for more than 440,000 deaths each year. Approximately 10% of these deaths are attributable to exposure of non-smokers to secondhand smoke (SHS). Residents living in public multi-unit housing (MUH) are at excess risk for SHS exposure compared to the general population. On November 30, 2016, the U.S. Department of Housing and Urban Development (HUD) passed a rule requiring all public housing agencies to implement smoke-free housing (SFH) policies in their housing developments by July 30, 2018.

**Methods:**

As part of a larger natural experiment study, we designed a protocol to evaluate indoor SHS levels before and after policy implementation through collection of repeat indoor air samples in non-smoking apartments and common areas of select high-rise NYCHA buildings subject to the HUD SFH rule, and also from socio-demographically matched private-sector high-rise control buildings not subject to the rule. A baseline telephone survey was conducted in all selected buildings to facilitate rapid recruitment into the longitudinal study and assess smoking prevalence, behaviors, and attitudes regarding the SFH policy prior to implementation. Data collection began in early 2018 and will continue through 2021.

**Discussion:**

The baseline survey was completed by 559 NYCHA residents and 471 comparison building residents (response rates, 35, and 32%, respectively). Smoking prevalence was comparable between study arms (15.7% among NYCHA residents and 15.2% among comparison residents). The majority of residents reported supporting a building-wide smoke-free policy (63.0 and 59.9%, respectively). We enrolled 157 NYCHA and 118 comparison non-smoking households into the longitudinal air monitoring study and performed air monitoring in common areas. Follow up surveys and air monitoring in participant households occur every 6 months for 2.5 years. Capitalizing on the opportunity of this federal policy rollout, the large and diverse public housing population in NYC, and robust municipal data sources, this study offers a unique opportunity to evaluate the policy’s direct impacts on SHS exposure. Methods in this protocol can inform similar SFH policy evaluations elsewhere.

## Background

Tobacco remains a leading cause of preventable death in the U.S., responsible for more than 440,000 deaths each year. Approximately 10% of these deaths are attributable to exposure of non-smokers to secondhand smoke (SHS) [1, 2]. Among children, SHS exposure increases the risk for lower respiratory infections, middle ear infections, and the number and severity of asthma attacks [3]. Among adults, SHS exposure increases the risk of cancer, coronary artery disease, stroke, and serious respiratory problems. SHS exposure is also associated with a number of poor birth outcomes, including low birth weight, premature delivery, congenital defects, and sudden infant death syndrome [1].

Comprehensive smoke-free laws in workplaces and public spaces have played a key role in reducing SHS exposure in the U.S. [4–7] Since the introduction of these policies, the prevalence of SHS exposure, as measured by cotinine levels among non-smokers nationally, fell from 87.5% in 1988 to 25.2% in 2014 [8]. Other factors associated with these policies that have contributed to the decline in exposure include increased adoption of voluntary smoke-free home policies, and decreases in smoking prevalence [9, 10]. Despite this progress, reductions in SHS exposure have stalled in recent years [8], and 58 million non-smokers in the U.S. are still exposed to SHS, primarily at home [4].

On November 30, 2016, the U.S. Department of Housing and Urban Development (HUD) passed a rule requiring all public housing agencies to implement smoke-free housing (SFH) policies in their developments within 18 months of rule execution – or by July 30, 2018 [11]. The regulation requires that any housing authorities administering low-income conventional public housing prohibit the use of tobacco products like cigarettes, cigars, pipes, and hookah in residential units, indoor common areas, and within 25 ft of buildings. This policy will potentially have long-lasting effects on the health of millions of Americans [12].

Residents living in public housing are at excess risk for SHS exposure, in part because they are predominantly lower-income minorities, groups which tend to have higher smoking rates compared with the general population [13–16]. At the same time, low income and racial/ethnic minority families are more likely to live in multi-unit housing (MUH), a physical environment that facilitates the smoke accumulation and places residents at elevated risk for involuntary exposure to SHS compared to residents living in detached housing [16]. A majority of public housing residents live in MUH and are, therefore, at higher risk of exposure compared to the general population [17–19]. Indeed, children of non-smoking families living in MUH have 45% higher cotinine levels than children who live in non-smoking single-family homes [20]. Given the significant negative health impacts and growing socioeconomic disparities related to SHS exposure, as well as evidence that most SHS exposure occurs at home, there is a strong scientific rationale for expanding smoke-free policies to include public housing [14, 19].

Only a few studies have measured changes in SHS exposure before and after implementation of housing-wide bans [21, 22]. While several studies have examined the post-implementation impacts of SFH on self-reported SHS exposure [21, 23–25], as of early 2019 only researchers in Boston and Philadelphia have evaluated a smoking ban in public housing apartments using state-of-the-art objective measures of SHS exposure such as airborne nicotine and particulate matter less than 2.5 μm in aerodynamic diameter (PM2.5) concentrations [22, 26–29]. An early cross-sectional study in Boston (*n* = 32 non-smoker apartments) found significantly lower PM2.5 concentrations and airborne nicotine levels in buildings with smoke-free policies compared with buildings without a similar ban [28]. However, results from these studies were inconsistent with respect to whether such policies result in detectable reductions in airborne nicotine or particulate matter (PM) in the homes of non-smoking residents, in part due to the challenges of small sample sizes and limited durations of post-policy follow up [22, 27, 29]. To foster harmonization of methods across studies examining similar policies, we present our detailed protocol here.

## Methods/design

### Study goals

This paper describes a protocol to evaluate the impact of smoke-free housing laws on SHS exposure by measuring airborne nicotine and PM2.5 in non-smoking apartments and common areas of New York City Housing Authority (NYCHA) buildings with smoke-free policies compared to matched buildings without policies. NYCHA is the largest housing authority in the United States; its more than 400,000 residents comprise approximately 15% of all public housing residents in the country [30]. To build upon existing studies, we designed this study to monitor the impact of the 2018 policy change in a large sample of apartments for up to 2.5 years post-policy. We also developed field protocols for counting observed cigarette butts and indoor smoker sightings in the common areas of selected buildings, also described here.

### Study design

Using a 3-year natural experiment study design, the research team designed a protocol to evaluate indoor SHS levels before and after policy implementation (tracking every 6 months for 2.5 years post-policy) in two arms. Per protocol, we planned to enroll and do repeat indoor air sample collection on 150 non-smoking apartments, and four common areas of 10 select high-rise NYCHA buildings in one arm, and compare them to a second arm of 150 apartments and common areas from 11 demographically matched private-sector high-rise buildings comprised of large numbers of tenants who receive subsidized housing vouchers known as Section 8 (‘Section 8 buildings’). Section 8 comparison buildings did not have smoke-free policies in place at enrollment into the study, nor did any have imminent plans to roll out smoke-free policies yet share similar demographic characteristics with the NYCHA buildings selected.

**Fig. 1:**
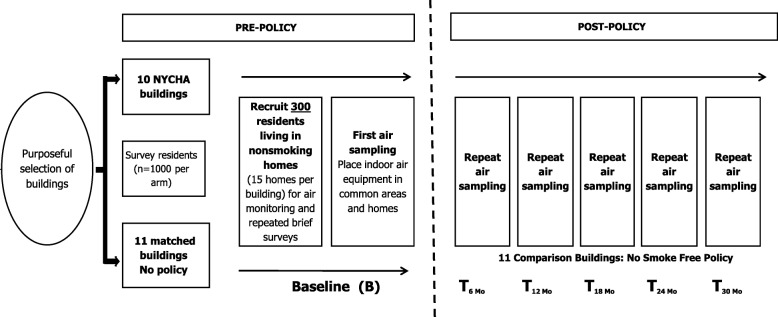
Study design of the smoke-free housing evaluation study’s air monitoring component

Data collection began in early 2018 and will continue through 2021, with a total of 1 pre-policy and 5 post-policy time points. Figure 1 illustrates the study design that began with the purposeful selection of 10 NYCHA and 11 matched comparison high-rise buildings (each > 15 floors). Table 1 illustrates the estimated selection of sites (household and common areas to be monitored) for this study. Before the implementation of the smoke-free policy, we measured SHS exposure for 7 days in each building’s common areas (e.g., hallways and stairwells) and in non-smoking households (target = 15 households per building), and we plan to measure SHS prospectively, taking a 7-day measurement every 6 months, over a 2.5-year period post-policy implementation. Methods used for pre-policy air monitoring are described below; post-policy measurements will follow the same protocol.

**Table 1:** Air monitoring sites and schedule per wave

	# of buildings	Target # of HH^a^ per bldg	Total # of HH	# of common areas per bldg	Total # of common areas	Total # of sites
Month 1	5 Intervention	15	75	4	20	95
	5 Comparison	15	75	4	20	95
Month 2	5 Intervention	15	75	4	20	95
	6 Comparison	15	90	4	24	114
					Total:	399

^a^*HH* households

### Study population and building selection

**Fig. 2:**
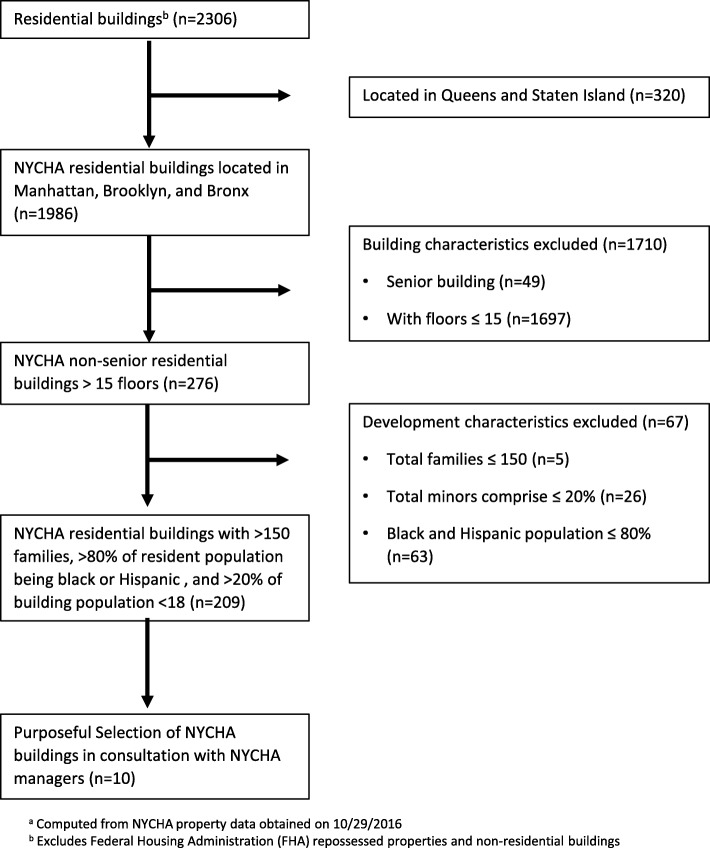
Process for Selecting NYC Housing Authority (NYCHA) Buildings into the Smoke Free Housing Policy Evaluation Study^a^

Study buildings (NYCHA, comparison) were eligible for inclusion in this study if they met the following criteria based on previous literature and expert opinion, designed to ensure homogeneity within and between comparison groups: (1) high-rise building (> 15 floors); (2) large resident population (> 150 families); (3) > 80% of the resident population being black or Hispanic (mirroring racial/ethnic distribution found in most NYCHA buildings); (4) > 20% of building population < 18 years old; and (5) located in Manhattan, Brooklyn, or the Bronx. Figure 2 illustrates the selection algorithm resulting in the final eligible buildings from which we selected our sample of intervention buildings. Once eligible NYCHA buildings were identified (*n* = 208), we worked collaboratively with NYCHA management officials to purposefully select buildings with larger population sizes and no imminent plans to be privatized. We also purposefully selected a subset of Section 8 buildings as the comparison group, matched to NYCHA demographically and in building structure.

### Participant recruitment

The study was launched by conducting a telephone survey of adult residents living in selected buildings to (1) recruit non-smoking households into the longitudinal air monitoring study; and to (2) obtain baseline information in both intervention and comparison building arms regarding smoking prevalence, prevalence of household smoking policies, and attitudes and knowledge regarding the smoke-free housing policy prior to policy implementation. All households in selected buildings with telephone numbers on file were initially approached, and adults aged 18 and older who lived in selected households and spoke either English or Spanish were eligible for survey participation. Only one member of a household was asked to respond. Households were eligible for inclusion in the longitudinal home air monitoring if the respondent indicated that it was a non-smoking household (defined as no household members are currently known to smoke cigarettes or use other tobacco products including e-cigarettes, which can contribute to airborne nicotine and PM2.5) [31, 32]. Participants also needed to confirm not living on the first three floors of a building to avoid ambient outdoor SHS [33].

The telephone survey was conducted by the staff of the CUNY Baruch Center for Survey Research (BCSR), who called documented telephone numbers associated with apartments in selected buildings located above the third floor in a two month time period using computer-assisted telephone interviewing techniques. Telephone numbers were provided by the NYCHA Department of Research and Management Analysis from the Tenant Data System (TDS). The brief 10-min survey was pilot-tested using cognitive testing methods with 15–20 NYCHA residents in non-selected buildings pre-implementation. Up to 8 call attempts were made per telephone number during weekdays, evenings, and weekends, using all available landline and mobile telephone numbers on file per household. Door-to-door recruitment was used to augment response rates using trained staff. Prior to the survey, we estimated 64–104 non-smoking households would complete the survey per building, depending on building size and building-specific smoking rates, with a goal to enrolled 15–20 households into the longitudinal study. These households were invited to participate in the longitudinal air-monitoring component and offered $50 per completed wave (6 waves; up to $300 compensation).

### Survey measures

In addition to facilitating rapid recruitment into the longitudinal study, survey data were used to develop a detailed profile of buildings. Survey measures included: 1) Building: proportion of households with current smokers; proportion of households with smoke-free policy; reported frequency of smelling SHS [17]; and proportion of respondents reporting feeling safe in their building and the surrounding neighborhood; 2) Household: proportion of household members who smoke; 3) Individual: demographics; current smoking prevalence (cigarette, e-cigarette) and use of other tobacco products; knowledge about SHS and support for smoke-free policy [34]; and 4) Interpersonal: perceived social norms about smoking in homes and common areas; social cohesion; and lodging complaints [34, 35].

### Study outcomes

#### Nicotine concentration levels

Numerous experts have emphasized the superiority of air quality monitoring, in particular, air nicotine and PM2.5, over biological cotinine as a measure of SHS exposure in housing [26, 36–38]. Cotinine measurements/levels are not specific in their assessment of SHS exposure obtained from the home, are subject to racial/ethnic absorption variability, are costly, and are not always acceptable, particularly among parents of children [39–41].

Only nicotine is specific to tobacco smoke [41, 42] and therefore, in this exposure assessment study, we selected airborne nicotine as the primary outcome. Prior to policy rollout, we measured airborne nicotine over a 7-day sampling period using passive bisulfate-coated filters placed in the private households of enrolled non-smokers and in the common areas in the selected high-rise buildings. Passive samplers were prepared and analyzed at Johns Hopkins University (JHU) Bloomberg School of Public Health using standard operating procedures established at Hopkin’s Secondhand Smoke Exposure Assessment Laboratory (http://www.shsmonitoring.org/analysis/lab/). Several studies have validated this method of monitoring nicotine as a reasonable, cost-efficient, and feasible assessment of SHS exposure [42, 43]. Monitors were sealed in an impermeable cassette until placed in participants’ apartments. Once unsealed, they were left in place for 7 days, after which they were resealed on-site by field staff, retrieved, and stored at room temperature until analysis. To assess the presence of nicotine in common areas of the buildings, samplers were placed in two randomly selected hallways from floors above the third floor and two randomly selected stairwells. The filters are placed in the same common area in each wave.

Filter blanks and replicates were included in the sampling design for quality control of the measurement of airborne nicotine by passive sampling. Field blanks (handled the same as the building samples but remain sealed) and replicates (duplicate samplers placed next to each other at a single site) comprised approximately 5% of all samples. The location of each sample within the building was recorded and placed according to guidelines developed by the SHS Assessment Laboratory at JHU (1–2 m from the floor; at least 1 m away from window or ventilation system; preferably not in ‘dead zones’ such as a corner; and minimally visible) [44]. Identical methodology will be used at each wave of data collection.

#### PM concentrations

In addition to airborne nicotine, airborne PM concentrations were monitored as a secondary outcome, using AirBeam monitors, a novel low-cost particle sensor [45] that has been shown to be accurate and precise for monitoring PM concentrations [46]. The Airbeam data were captured in real time and saved by a smartphone app (Aircasting) at 1 min intervals for the 7-day period. The Airbeams were placed alongside the passive nicotine sampler at 1 m above the floor in the living room or den of each residence. The average weekly indoor PM2.5 concentration was reported as μg/m^3^. To increase the rigor of the AirBeams measurements, AirBeam sensors are individually pre-calibrated with cigarette smoke, in a dynamic exposure chamber, against a more advanced light scattering PM_2.5_ monitoring instrument – a DataRam PDR 1500 (Thermo Environmental Instruments), prior to use and at 12 month intervals, humidity and temp correction was not performed [47]. Using the continuous 1-min interval measurements for both instruments, calibration curves were generated for each Airbeam unit [48]. In a subset of homes, we also cross-validated the AirBeam PM data by co-locating a DataRam PDR 1500 (calibrated annually by the manufacturer, Thermo-Scientific). An Identical methodology will be used at each wave of data collection with consideration for seasonality.

#### Butt count and active smokers sighting

Air quality outcomes were supplemented with field observational data. Quantifying cigarette butts is an approach that has previously been implemented as an observational assessment of smoke-free policy compliance [49–56]. Enumeration of cigarette butts and smokers observed in indoor common areas occurred twice pre-policy and will occur during each subsequent wave of air monitoring data collection in all 21 buildings selected for the study. During each of these visits, environmental field staff makes rounds to count discarded cigarette butts inside NYCHA and Section 8 buildings in hallways and stairwells (4 hallways per building and bottom 10 floors of 2 stairwells), and document the number of indoor smokers they observe during this process, usually taking 30 min. Field staff selected stairwells by convenience if more than two stairwells existed within a building. Hallways for this method were randomly selected by central coordinating staff prior to each wave. The first round of butt count and active smokers sighting measurements occurred when air monitors were beginning to be placed in a building (e.g. first visit of the wave). The second round of hallway and stairwell butt count and active smokers sighting measurements for that wave occurred at the beginning of month two. All data were documented on a dedicated form via electronic case report using REDCap or initially on paper, if necessary. We used this quantitative approach in select public housing authority buildings as an ancillary method to evaluate adherence to the HUD SFH policy over time. The counting procedures used here were designed based on the Tobacco-Free Compliance Assessment Tool (TF-CAT) [57].

### Statistical analysis

#### Nicotine and PM concentration levels

Extracted nicotine filters are analyzed by gas chromatography and the airborne concentration of nicotine calculated by dividing the μg nicotine/filter by the volume of air sampled [45]. The sampling rate has been validated at 25 mL/min [43]. Air nicotine concentration is measured in units of μg/m^3^ and samples below the limit of detection (0.033 μg/m^3^) are reported as ½ the limit of detection [58], assuming constant airflow in deployment [22].

PM concentrations measured by the Airbeams were adjusted by applying the coefficients of the individual calibration curves/formulas generated for each Airbeam.

For each wave of data collection, general descriptive statistics (frequencies, means, medians, standard deviations, inter-quartile ranges) are calculated for measures of airborne nicotine concentration and PM levels, characterizing SHS exposure in the indoor environments of buildings within each comparison group over time. The number of discarded tobacco products and smokers sightings are summed, averaged over the 2 observation occasions and then analyzed by stairwells and hallways. The primary and secondary outcomes will be analyzed using a difference in difference (DID) approach for repeated measures and the model will include fixed effects for (1) intervention vs comparison buildings, (2) post-implementation time-periods (5-time points, 2.5 years) and (3) “intervention*time” interaction, adjusting for within-apartment, within-building, and seasonal correlations, as needed [59–61]. The policy intervention effect will be estimated by interaction coefficients over time, allowing flexibility to assess immediate vs delayed effects. Given expected skewed nicotine concentration distributions, we will use a log transformation.

#### Sample size calculation

We designed the study to have adequate power (≥0.80) to detect at least 0.26 μg/m3 difference from baseline to 2.5-years post-policy implementation between the intervention and control buildings on change in the primary outcome (airborne nicotine concentration). We chose this conservative difference level based on published findings from an evaluation of a smoking-free policy in Boston public housing, although meta-analyses have identified larger effects [22, 36]. Accumulating literature shows there is no safe level of SHS and that smoke-free legislation has been associated with improved health outcomes, thus this effect size is expected to have high public health relevance [62, 63]. The change estimate of 0.26 μg/m3 nicotine levels relates to SHS in common building areas [27], but we anticipate a greater change in non-smoking apartments [17, 28]. We assumed a pooled apartment-to apartment standard deviation for the primary outcome of 0.8–2 μg/m3 (corresponding to an effect size of 0.33–0.50 standard deviation units) [27] and an intraclass correlation (ICC) ≤0.02 within buildings of and ≤ 0.1 over time [64].

For the secondary analyses (PM concentration level), our design has adequate power to detect relatively small effect sizes (0.20 standard deviation range for continuous outcomes and 10–15% difference in proportions), based on the assumption of relatively low ICC (≤0.02). Lastly, we chose the number of buildings vs. apartments in this study to maximize power taking into account logistics. Power is maximized with our choice of a larger number of buildings relative to a number of apartments per building [65]. Sample size decisions for the initial telephone survey were made to assure adequate recruitment into the longitudinal household air-monitoring component, but statistical power consideration was also given to ensure adequate power to estimate arm-specific smoking prevalence for a range of plausible estimates (15–25%) with a margin of error of < 2%.

### Study enrollment results

The baseline survey was completed by a total of 559 NYCHA residents and 471 residents from comparison buildings residing above the third floor in the selected NYCHA and Section 8 buildings. Using the American Association for Public Opinion Research (AAPOR) standard definition for response rate in unknown eligibility [66], the response rate was 35.3% among eligible residents in selected NYCHA buildings and 32.1% among eligible residents in Section 8 buildings. The estimated smoking prevalence at baseline was 15.7% in NYCHA buildings and 15.2% in Section 8 buildings. Among NYCHA survey participants, 62.3% reported smelling cigarette smoke in their home, coming from another apartment or from both outside of the building or hallway/stairwell in the past year, and 77.3% reported seeing people smoke in common areas. In comparison, 57.2% of Section 8 participants reported smelling cigarette smoke in their home, coming from another apartment or from outside in the past year, and 55.6% reported seeing people smoke in common areas. The majority of NYCHA and Section 8 participants reported supporting a building-wide smoke-free policy (63.0 and 59.9%, respectively).

From the survey, 157 NYCHA and 118 Section 8 non-smoking households were enrolled in the longitudinal air monitoring study. Recruitment for this phase of the study took place between April and July 2018 for NYCHA participants, and between July and November 2018 for Section 8 participants. Table 2 shows demographic characteristics of our baseline sample, the median (SD) age of participants who consented to be in the study was 52.1 (±16.4) among NYCHA participants and 55.7 (±14.4) among Section 8 participants. The majority of NYCHA study participants (72.6%) were female, and identified as Hispanic/Latino (51.9%) or Black (39.2%); a similar demographic profile was found among Section 8 participants (75.2% female; 50.0% Hispanic/Latino; and 40.0% Black). Among NYCHA participants, nearly half (43.5%) reported having children living in the home and similar results were found among Section 8 participants (41.7%). Among NYCHA participants, 42.3% of the residents reported a household member with an asthma diagnosis, compared to 38.6% of Section 8 participants.

**Table 2:** Demographic Characteristics in the Longitudinal Study

Demographics	NYCHA (*n* = 157)	Section 8 (*n* = 118)
Enrolled By Building, Range	14–19	5–18
Age (Mean, SD)	51.0 (16.8)^a^	56.3 (14.4)^a^
Age (N, %)^a^		
20–34	46 (29.3)	22 (18.6)
35–64	80 (51.0)	65 (55.1)
65+	31 (19.7)	31 (26.3)
Gender (N, %)		
Male	42 (26.8)	28 (24.6)
Female	114 (72.6)	86 (75.4)
Other	1 (0.6)	
Race/Ethnicity (N, %)		
Non-Hispanic Black	61 (38.9)	44 (40.0)
Asian	2 (1.3)	0 (0.0)
Hispanic or Latino	82 (52.2)	55 (50.0)
White	2 (1.3)	1 (0.9)
More than one race	3 (1.9)	7 (6.4)
Unknown	6 (3.8)	2 (1.8)
American Indian/ Alaska Native	1 (0.6)	1 (0.9)
Language (N, %)		
English	112 (71.3)	83 (73.4)
Spanish	45 (28.7)	30 (26.6)
Number of Adults in the Home (N, %)		
1	63 (40.9)	46 (40.0)
2	46 (29.9)	43 (37.4)
3 or more	45 (29.2)	26 (22.6)
Number of Children in the Home (N, %)		
0	87 (56.5)	67 (58.3)
1	30 (19.5)	22 (19.1)
2	21 (13.6)	17 (14.8)
3 or more	16(10.4)	9(7.8)

^a^information is available only from 141 NYCHA and 101 Section 8 Households

## Discussion

Capitalizing on the opportunity to perform a natural experiment to study the impact of this landmark federal policy and the exceptionally large and diverse public housing population in NYC, this study offers an opportunity to rigorously measure the policy’s direct impacts on SHS exposure. Findings will be used to develop guidance for implementing SFH policies in MUH settings nationally.

## Data Availability

The datasets that will be analyzed for this study will be available from the corresponding author on reasonable request, once final results are published.

## Abbreviation

HUD: Department of Housing and Urban Development
MUH: Multi-unit housing
NYCHA: New York City Housing Authority
PM: Particulate matter
SFH: Smoke-free housing
